# Plasma NfL, GFAP, amyloid, and p-tau species as Prognostic biomarkers in Parkinson’s disease

**DOI:** 10.1007/s00415-024-12669-7

**Published:** 2024-09-09

**Authors:** Andrea Pilotto, Nicholas J. Ashton, Alessandro Lupini, Beatrice Battaglio, Cinzia Zatti, Chiara Trasciatti, Stefano Gipponi, Elisabetta Cottini, Ilaria Grossi, Alessandro Salvi, Giuseppina de Petro, Marina Pizzi, Antonio Canale, Kaj Blennow, Henrik Zetterberg, Alessandro Padovani

**Affiliations:** 1 Neurology Unit, Department of Clinical and Experimental Sciences, University of Brescia, P.Zzale Spedali Civili, 1, 25123 Brescia, Italy; 2 Department of Continuity of Care and Frailty, Neurology Unit, ASST Spedali Civili Hospital, Brescia, Italy; 3 Neurobiorepository and Laboratory of Advanced Biological Markers, University of Brescia and ASST Spedali Civili Hospital, Brescia, Italy; 4 Department of Psychiatry and Neurochemistry, Institute of Neuroscience and Physiology, The Sahlgrenska Academy, University of Gothenburg, Gothenburg, Sweden; 5 Wallenberg Centre for Molecular and Translational Medicine, Department of Psychiatry and Neurochemistry, Institute of Neuroscience and Physiology, The Sahlgrenska Academy at the University of Gothenburg, Gothenburg, Sweden; 6 King’s College London, Institute of Psychiatry, Psychology and Neuroscience, Maurice Wohl Clinical Neuroscience Institute, London, UK; 7 NIHR Biomedical Research Centre for Mental Health and Biomedical Research Unit for Dementia at South London and Maudsley NHS Foundation, London, UK; 8 Division of Biology and Genetics, Department of Molecular and Translational Medicine, University of Brescia, Brescia, Italy; 9 Division of Pharmacology, Department of Molecular and Translational Medicine, University of Brescia, Brescia, Italy; 10 Department of Statistical Sciences, University of Padova, Padua, Italy; 11 Clinical Neurochemistry Laboratory, Sahlgrenska University Hospital, Mölndal, Sweden; 12 Paris Brain Institute, ICM, Pitié-Salpêtrière Hospital, Sorbonne University, Paris, France; 13 Neurodegenerative Disorder Research Center, Division of Life Sciences and Medicine, Department of Neurology, Institute On Aging and Brain Disorders, University of Science and Technology of China and First Affiliated Hospital of USTC, Hefei, People’s Republic of China; 14 Department of Neurodegenerative Disease, UCL Institute of Neurology, London, UK; 15 UK Dementia Research Institute at UCL, London, UK; 16 Department of Old Age Psychiatry, Institute of Psychiatry, Psychology, and Neuroscience, King’s College London, London, UK; 17 Hong Kong Center for Neurodegenerative Diseases, Hong Kong, People’s Republic of China; 18 Wisconsin Alzheimer’s Disease Research Center, University of Wisconsin School of Medicine and Public Health, University of Wisconsin-Madison, Madison, WI USA; 19 Brain Health Center, University of Brescia, Brescia, Italy

**Keywords:** Parkinson’s disease, Neurofilament light chain, GFAP, Phosphorylated tau, Progression, Plasma biomarkers

## Abstract

**Introduction:**

The prognostic role of plasma neurofilament light chain (NfL), phospho-tau, beta-amyloid, and GFAP is still debated in Parkinson’s disease (PD).

**Methods:**

Plasma p-tau181, p-tau231, Aβ1-40, Aβ1-42, GFAP, and NfL were measured by SIMOA in 136 PD with 2.9 + 1.7 years of follow-up and 76 controls. Differences in plasma levels between controls and PD and their correlation with clinical severity and progression rates were evaluated using linear regression analyses.

**Results:**

Patients exhibited similar distribution of plasma biomarkers but higher P-tau181, P-tau231 and lower Aβ1-42 compared with controls. NfL and GFAP correlated with baseline motor and non-motor severity measures. At follow-up, NfL emerged as the best predictor of progression with marginal effect of GFAP and p-tau181 adjusting for age, sex, disease duration, and baseline motor severity.

**Conclusion:**

The present findings confirmed plasma NfL as best predictor of progression in PD, with a marginal role of p-tau181 and GFAP.

## Introduction

Parkinson’s disease (PD) is a heterogeneous neurodegenerative disorder characterized by different rates of motor progression and responses to treatment. Several demographic and clinical features have been associated with worse progression in PD in prospective cohorts, including older age, male sex, the akinetic-rigid phenotype, and the presence of autonomic dysfunction [1–4].

It has recently been demonstrated that cerebrospinal fluid (CSF) and plasma neurofilament light chain (NfL) represent the most effective blood marker for predicting disease progression in several neurodegenerative disorders, including Parkinson’s disease (PD) [5–8]. Moreover, glial fibrillary acidic protein (GFAP), a marker of glial activation, has been linked to disease severity and poorer progression in Alzheimer’s disease (AD), frontotemporal dementia (FTD), and the alpha-synucleinopathy dementia with Lewy bodies (DLB) [9–11]. Several large studies suggested that plasma phosphorylated tau (p-tau) and amyloid species may serve as highly reliable markers for identifying Alzheimer’s disease (AD) even in its early stages [12–16]. P-tau species, in particular p-tau181, have been identified as a promising avenue for detecting AD co-pathology and have been shown to possess prognostic value in alpha-synucleinopathies, as recently evidenced in DLB [17, 18]. Nevertheless, the prognostic value of plasma biomarkers in PD remain a topic of debate, and only a limited number of longitudinal findings are currently available in this field.

In this study, we hypothesize that a panel of plasma markers, including p-tau, amyloid species, and GFAP, might increase the known diagnostic ability of NfL to stratify PD patients and predict disease progression over time. To this end, we first compared these markers in PD and age-matched controls and evaluate the possible association with different severity measures at baseline. Furthermore, we evaluated the ability of single and multiple markers to predict motor and non-motor disease progression in PD, adjusting for the effect of baseline severity variables.

## Methods

### Patient’s selection

Consecutive patients with a clinical diagnosis of PD [19] were evaluated at the outpatient Movement disorder Clinic, Neurology Unit at the University of Brescia, North of Italy. It serves as a tertiary referral center for neurodegenerative disorders and it follows-up approximately 800 subjects with PD on a regular basis. Healthy controls (HC) were selected among patients’ caregivers. This study was approved by the local ethics committee (NP 1471, DMA, last version on December the 7th 2020) and was in conformity with the Helsinki Declaration. Informed consent was obtained from all participants at blood sampling. Levodopa equivalent daily dose (LEDD) was calculated according to standard conversion [20] and the diagnosis was supported by levodopa/dopaminergic response and at least 2 years of clinical follow-up. Only clinically established PD patients [21] were included.

All patients underwent routine blood analyses and magnetic resonance imaging to exclude prominent cortical or subcortical infarcts or brain/iron accumulation or atypical parkinsonian disorders. The following exclusion criteria were applied: (1) dementia at baseline; (2) atypical parkinsonism, at baseline or during follow-up; (3) prominent cortical or subcortical infarcts in structural imaging; (4) other neurologic disorders or medical conditions potentially associated with cognitive deficits; (5) bipolar disorder, schizophrenia, history of drug or alcohol abuse, or impulse control disorder; (6) negative nigrostriatal dopaminergic imaging; (7) recent traumatic events or acute fever/inflammation; (8) kidney disease.

### Clinical assessment

At baseline, standardized neurological examination was performed, including the Movement Disorder Society-Unified Parkinson Disease Rating Scale (MDS-UPDRS) [22] and Hoehn and Yahr stage (H and Y) assessment [23]. The following disability milestones were evaluated at baseline and after follow-up for each patient: gait dependency (unable of walking unassisted), recurrent falls (more than 1 fall per month), motor fluctuations (OFF state and/or dyskinesia), and dementia (cognitive impairment causing dependency in ADL).

All patients included in the analyses underwent a clinical and follow-up for at least 2 years and up to 5 years (average follow-up: 2.9 years). The patients were stratified according to the mean annual change in MDS-UPDRS-III; fast progressor were defined as subjects with an MDS change higher than one standard deviation in the cohort (UPDRS-III of > 2 vs ≤ 2 points, respectively) independently from levodopa equivalent daily dose adjustment. The annual changes in MDS-UPDRS and development of disability milestones were considered as linear and dichotomic targets for biomarkers analyses.

### Biochemical analyses

At the time of assessment, approximately 10 mL venous blood was collected in tubes containing sodium ethylenediaminetetraacetic acid (EDTA) from each subject. Participants were required to fast for at least 2 h prior to collection. The blood samples were centrifuged at 2000 × *g* at 4 °C for 8 min within 2 h of collection. Plasma supernatant was collected, divided into aliquots, and frozen at − 80 °C until further use. NfL, p-tau181 and p-tau231, Aβ1-40, Aβ1-42, and GFAP concentrations were measured at the Clinical Neurochemistry Laboratory, Sahlgrenska University Hospital, Mölndal, Sweden. Biomarkers concentrations were measured using the Simoa platform (Quanterix, Billerica, MA). For p-tau231, an in-house assay was used as previously described [14]. The other markers were measured using commercially available assays (Quanterix, Billerica, MA). Samples were randomized, blinded, and measured in duplicate using one batch of reagents from the same lot in one round of experiments. Intra-assay coefficients of variation were below 10%. Individuals exhibiting plasma values exceeding five standard deviations of the mean for the entire sample were excluded from the study analysis. Additionally, cases with missing values or outliers were excluded.

### Statistical analyses

Differences in demographic features between PD and HC were assessed with t-test for independent samples or Chi-square test for continuous and categorical variables, respectively. Differences within biomarkers levels between PD and HC were evaluated using Student T test for independent variables. Partial correlation analyses adjusted for the effect of age, sex, and disease duration was applied to test significant correlations between biomarkers values and clinical variables (MDS-UPDRS-III, LEDD, and H and Y). Differences in biomarkers level between PD with normal and fast progression were assessed in univariate analyses adjusted for the effect of age, sex, and disease duration. The discriminative ability of the single biomarkers in predicting fast and slow progressors was evaluated through an overall accuracy analysis utilizing the area under the curve (AUC) of a receiver-operating characteristic curve (ROC) and the positive and negative predictive values. The between MDS-UPDRS-III and plasma biomarkers levels was additionally evaluated using a linear regression model adjusted for the effect of age, sex, disease duration, and a principal components regression where all the plasma biomarkers have been decomposed with a principal component analysis, later used as matrix of covariate in a linear model with MDS-UPDRS-III as response.

Differences in time-dependent disability milestones between patients with normal and abnormal biomarkers levels were assessed with Cox regression corrected for age at sampling, disease duration, and sex. Significance was set at *p* < 0.05 for all the analysis.

## Results

### Recruitment, and clinical and cognitive baseline features

**Fig. 1:**
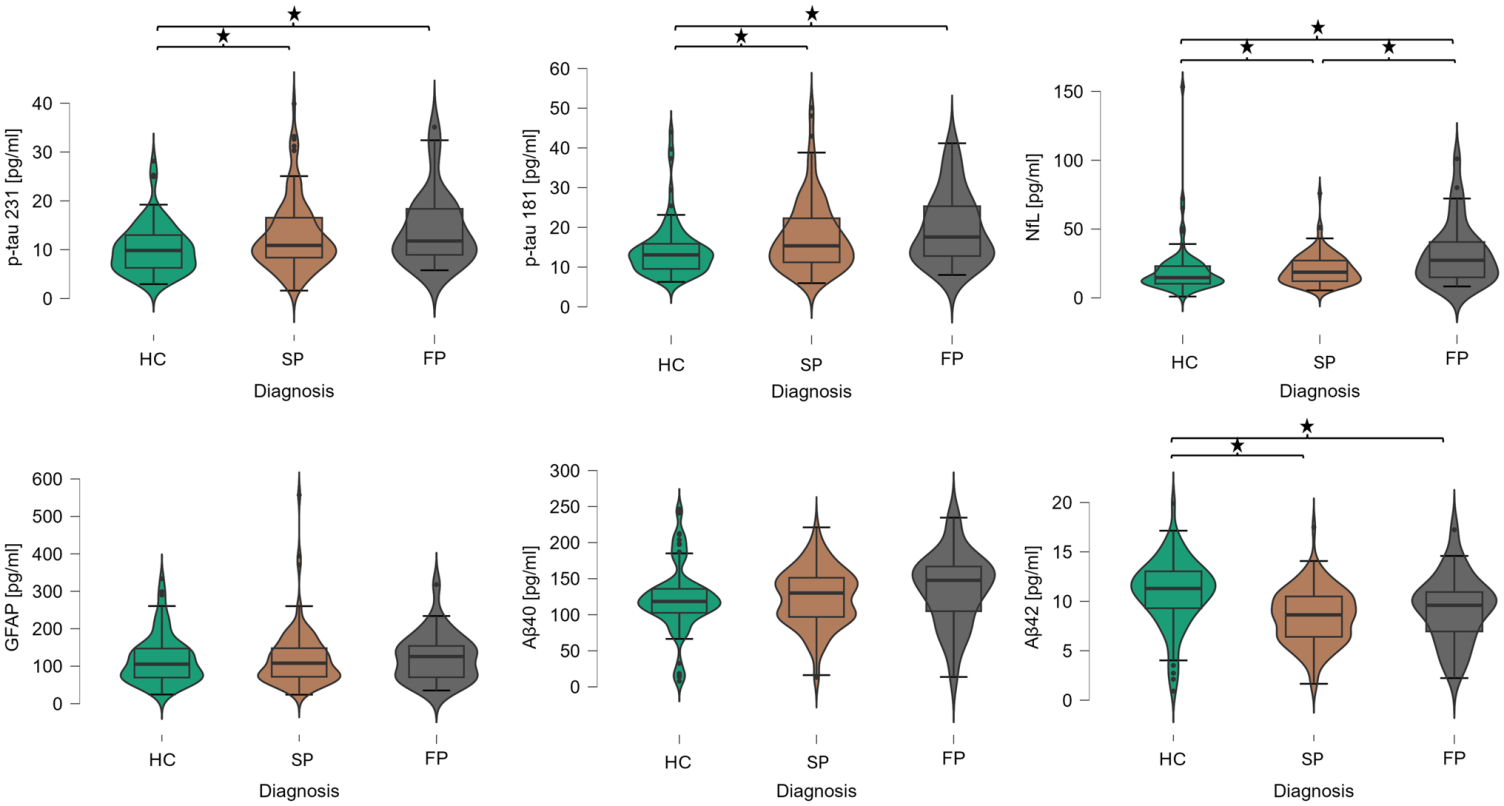
Correlation matrix including age at onset, disease duration, and the biomarkers analyzed in the cohort. p-tau181, phosphorylated Tau 181, p-tau231, phosphorylated Tau 231, Aβ1-40, beta-amyloid 1–40; Aβ1-42, beta-amyloid 1–42; *GFAP* Glial Fibrillary Acidic Protein, *NfL* neurofilament light chain; *HC* healthy controls; *SP* PD with slow motor progression; *FP* PD with fast motor progression

Out of 190 patients with a clinically confirmed diagnosis of Parkinson’s disease under dopaminergic treatments screened, 136 entered the study (mean age 69.3 ± 9.8 years, mean disease duration 6.5 ± 5.0 years); 78 age-matched controls were included in the analysis (supplementary Fig. 1 for the inclusion flowchart). Table 1 shows the demographics and clinical baseline characteristics and biomarkers level of controls and PD stratified according to the longitudinal follow-up in fast and slow progressors. Compared with HC, PD patients showed higher levels of p-tau231 and p-tau181 and lower levels of Aβ1-42 levels. PD patients with fast motor progression exhibited higher NfL levels compared to both HC and PD with normal motor progression. In the whole cohort and specifically in PD, all the markers correlated with age at sampling and disease duration (except for GFAP) (Fig. 1).

**Table 1:** Demographical and biomarkers difference between HC and PD

	HC (*n* = 78)	PD normal progression (*n* = 105)	PD fast progression (*n* = 31)	*P*
Sex (F)	54 (69.2%)	53 (39.0%)	53 (39.0%)	**< 0.001**^**x**^
Age at blood sample	69.4 ± 7.9	68.5 ± 10.0	72.0 ± 8.9	0.177
Disease duration, y	N.A	6.2 ± 4.8	7.6 ± 5.7	0.218
H and Y stage (*n*)	N.A	1 (29) 2 (59) 3 (14) 4 (2) 5 (1)	1 (6) 2 (16) 3 (6) 4 (2) 5 (1)	0.056
MDS-UPDRS-III	N.A	20.0 ± 12.4	19.1 ± 10.7	0.415
Annual MDS-UPDRS-III total score change	N.A	0.6 ± 2.0	6.5 ± 5.4	< 0.001
LEDD	N.A	482.0 ± 335.7	419.0 ± 215.2	0.326
Annual LEDD change	N.A	12.8 ± 103.3	66.8 ± 152.2	0.024
Plasma biomarkers				
p-tau 231 (pg/mL)	10.2 ± 5.1	12.9 ± 7.2	14.2 ± 7.4	**0.004**^**xy**^
p-tau 181 (pg/mL)	14.3 ± 6.9	17.7 ± 8.9	19.7 ± 8.9	**0.002**^**xy**^
Aβ1-40 (pg/mL)	121.5 ± 45.8	124.1. ± 39.4	135.9 ± 49.9	0.288
Aβ1-42 (pg/mL)	10.9 ± 3.4	8.4 ± 2.7	9.2 ± 3.4	**< 0.001**^**xy**^
GFAP (pg/mL)	117.7 ± 64.9	120.4 ± 75.8	123.0 ± 63.9	0.933
NfL (pg/mL)	20.1 ± 19.5	20.8 ± 10.9.2	23.4 ± 15.2	0.002^**yz**^

*H and Y* Hoehn and Yahr stages, *LEDD* levodopa equivalent daily dose expressed in mg; *MDS-UPDRS-III* Movement Disorder Society-Unified Parkinson’s disease Rating scale part III, *p-tau181* phosphorylated tau 181, *p-tau231* phosphorylated tau 231, *Aβ1-40* beta-amyloid 1–40, *Aβ1-42* beta-amyloid 1–42, *GFAP* glial fibrillary acidic protein, *N.A* not applicable, *NfL* neurofilament light chain, *y* years, *x* = HC vs PD normal progression, *y* = HC vs PD fast progression, *z* = PD normal progression vs PD fast progression

**Fig. 2:**
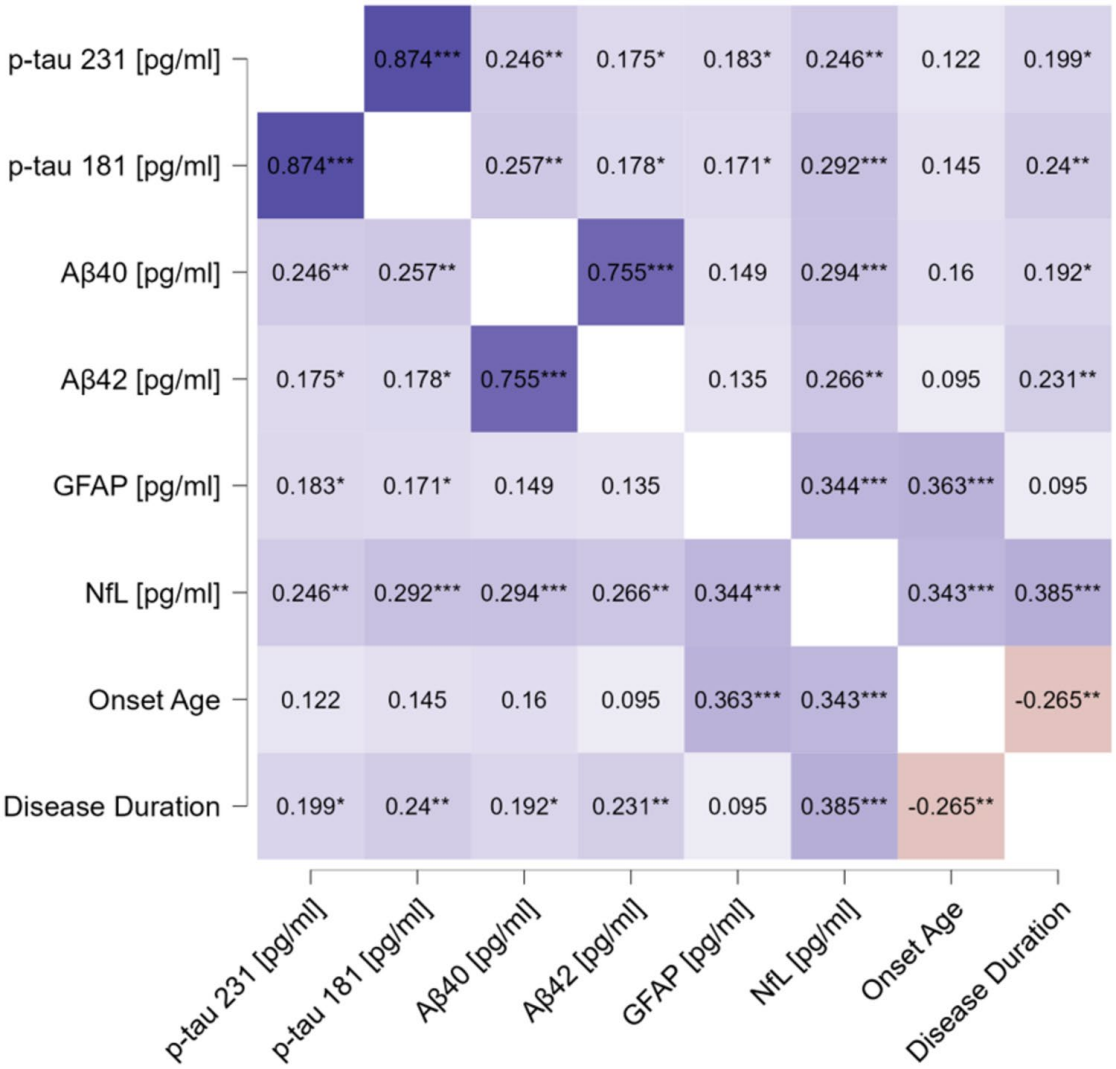
Cox regression analysis comparing development of dementia in patients with high NFL levels vs patients with normal NFL levels. *NfL* neurofilament light chain

In PD, plasma NfL and GFAP exhibited a positive correlation with total MDS-UPDRS-III scores at baseline in analyses adjusted for age, sex, and disease duration (*r* = 0.207; *p* = 0.007 and *r* = 0.163; *p* = 0.03, respectively). Both biomarkers additionally showed a positive correlation with Hoehn and Yahr stage (*r* = 0.433; *p* < 0.001 for NfL; *r* = 0.173; *p* = 0.036 for GFAP). The correlation between plasma biomarkers and cognition was evaluated in 96 PD patients with MoCA baseline assessment. In unadjusted analyses, higher levels of p-tau181 and NfL exhibited a negative correlation with MoCA (*r* = − 0.218, *p* = 0.048 and *r* = − 0.329, *p* = 0.001, respectively), whereas no correlation survived in corrected analyses. Moreover, upon analyzing the correlations between variables without adjusting for age and sex, significant positive associations were observed, particularly between NfL and GFAP (see Fig. 2 and supplementary Table 1).

### Correlation between plasma biomarkers and clinical variables at follow-up

The presence of milestones of disability and the annual change of MDS-UPDRS-III was evaluated in a clinical follow-up (average: 2.9 ± 1.7 years). In the PD group, 32 patients presented falls (14 at baseline, 18 during follow-up with a medium onset 1.3 years after baseline); 16 exhibited progressive inability to walk (2 at baseline, 14 during follow-up with a medium onset of 1.3 years after baseline) and 26 motor fluctuations (6 at baseline, 20 during follow-up) (see supplementary Table 2 for details). Patients with walking impairment (inability to walk unassisted) showed higher baseline mean levels of NfL compared with independent patients (37.5 ± 24.2 vs 21.5 ± 12.5, *p* < 0.001) with similar levels of other markers. The annual changes in MoCA were evaluated in a subset of 81 patients with at least three consecutive annual assessments. In partial correlation and linear correlation analyses adjusted for age, sex, education, and disease duration, no marker was found to be correlated with worsening of cognition status measured by annual rate change of MoCA.

**Fig. 3:**
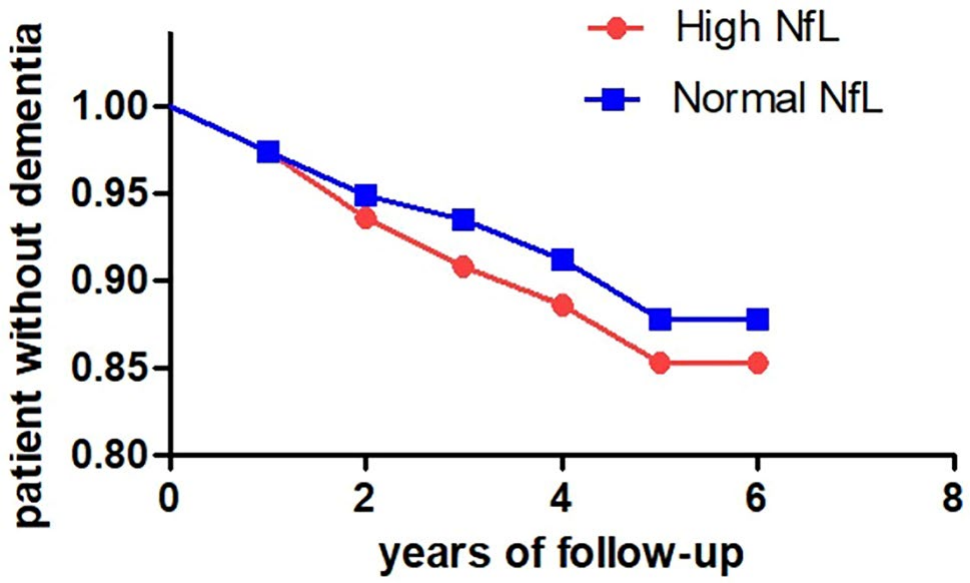
Differences in biomarkers levels in plasma between HC, and slow and fast PD progressors. p-tau181, phosphorylated tau 181, p-tau231, phosphorylated tau 231, Aβ1-40, beta-amyloid 1–40; Aβ 1–42, beta-amyloid 1–42; *GFAP* glial fibrillary acidic protein, *NfL* neurofilament light chain

The levels of plasma biomarkers did not differ between patients with/without falls and motor fluctuations (separately analyzed at baseline and during follow-up). Patients who developed dementia during follow-up (*n* = 30) exhibited higher levels of NfL compared with non-demented patients, when adjusted for age, sex, and disease duration (20.2 ± 12.1 vs 36.4 ± 22.1, *p* < 0.001). In Cox regression analysis corrected for sex, age, and disease duration, patients with abnormal plasma NfL levels had a higher risk of developing dementia during follow-up (*p* = 0.030) (Fig. 3). No other significant result was found in Cox regressions analyzing different biomarkers and disability milestones.

### Annual MDS-UPDRS and LEDD change and baseline plasma biomarkers

The relationship between annual change in MDS-UPDRS-III scores and plasma biomarkers was challenged in both partial correlation and linear regression models. NfL was the only plasma marker positively correlated with changes in MDS-UPDRS-III scores in both models (partial correlation *r* = 0.256; *p* = 0.003, linear regression model *p* = 0.02, T 2.2) (Table 2). The correlation between NfL and changes of UPDRS-III were confirmed after exclusion of PD patients with baseline falls (*r* = 0.223, *p* = 0.033); this was no longer significant in linear regression analysis (*r* = 0.139, *p* = 0.105) (Supplementary Table 3).

**Table 2:** Multivariable linear regression model for motor progression defined by annual MDS-UPDRS part III score changes including demographics, clinical baseline variables, and NfL levels

Model	*B*	Standard error	Beta	*T*	Sign
Constant	1.107	2.898		0.382	0.703
Age at sampling	0.017	0.044	0.039	0.382	0.382
Disease duration	0.096	0.097	0.112	0.987	0.325
Gender	− 0.828	0.722	− 0.094	− 1.147	0.254
NfL	0.067	0.029	0.233	2.265	0.025
MDS-UPDRS-III baseline	− 0.091	0.033	− 0.255	− 2.789	0.006
LEDD baseline	− 0.002	0.001	− 0.136	− 1.337	0.184
Falls baseline	1.914	0.836	0.189	2.289	0.024

In the first line, “B” refers to unstandardised coefficients, while “Beta” refers to the standardized coefficients

*LEDD* levodopa equivalent daily dose, *MDS-UPDRS-III* movement Disorder Society- Unified Parkinson disease Rating Scale, *NfL* neurofilament light chain

Linear combinations of NfL, p-tau181, p-tau231, Aβ1-40, Aβ1-42, and GFAP into six new variables obtained through principal component decomposition (after standardization) were used as regressors in a linear model with yearly delta MDS-UPDRS-III scores as response and correcting for age, sex, and disease duration. The only significant combination of variables (*p* = 0.02) was a combination of NfL and GFAP, confirming the association of these variables with delta MDS-UPDRS-III scores (Supplementary Table 4). A similar analysis on the original biomarkers (without standardizing their values) reports that the only significant combination of variables (*p* = 0.004) consisted of a combination of NfL and p-tau181 (Supplementary Table 5).

Figure 1 shows the different distribution of plasma biomarkers in patients with normal and fast progression, with NfL being the only marker able to differentiate PD patients with different rates of motor change (32.0 ± 22.6 vs 20.1 ± 19.5; *p* = 0.007). In ROC analyses, NfL demonstrated a positive predictive value of 45% and a negative predictive value of 85% for distinguishing between slow and fast progressors based on UPDRS, with an AUC of 0.65 (95% CI: 0.521–0.770) and a Youden optimal cutoff of 29.6 pg/mL.

Baseline LEDD score and annual changes were not correlated with any of the biomarkers at baseline, and none of them was able to differentiate different trajectories of LEDD changes across the years of follow-up.

## Discussion

This prospective study aimed to evaluate the clinical value of a panel of plasma biomarkers for stratifying PD patients and predicting disease progression over time. The findings showed a strong correlation of NfL and GFAP with motor severity at baseline, whereas p-tau181 correlated with cognition only in unadjusted analyses. At follow-up, NfL emerged as the strongest predictor of progression, with only a marginal effect of p-tau181 and GFAP using different statistical approaches.

These findings contribute to expanding the current knowledge regarding the clinical application of plasma biomarkers in PD by evaluating an extended panel of standard plasma markers using Simoa and conducting a clinical follow-up over 3 years. Several recent studies have supported the use of AD-related plasma biomarkers for early identification of AD due to their strong correlation with CSF species [16, 24, 25]. Phosphorylated tau species and GFAP have recently been highlighted as important prognostic markers in AD, atypical parkinsonism, and DLB [26, 27].

Furthermore, CSF AD-related biomarker patterns have been identified as important predictors of progression in PD—especially for cognitive measures [28–32] thus providing a strong rationale for the present study. Compared with age-matched controls, non-demented PD patients exhibited slightly higher levels of GFAP and p-tau species and lower levels of amyloid 1–42. This might indicate a concomitant AD-related pathology in a relevant percentage of PD [25, 29, 31, 33–36]. However, these trends might also indicate unspecific effects of PD pathology on some plasma biomarkers, whereas NfL—the most sensitive neuronal damage marker—appeared to have similar levels compared with controls [6, 7, 37, 38]. The baseline clinical correlation analyses demonstrated a robust correlation between motor severity and NfL, as well as GFAP. This is an intriguing finding, particularly considering the purported role of the latter biomarker in modulating nigrostriatal and cortical alpha-synuclein pathology [39, 40] and its purported utility as a prognostic marker in Asian ethnicity [41]. Conversely, cognitive function at baseline correlated with both NfL and p-tau181 only in unadjusted analyses, in line with the recent works focused on cognition [42].

The motor follow-up assessed with MDS-UPDRS scores, levodopa changes, and disability milestones confirmed NfL as the best predictor of progression for motor progression time, with only marginal impact for GFAP and p-tau181 in PCA-based models. NfL also emerged as the best marker for predicting conversion to dementia, whereas p-tau species, GFAP, and amyloid did not show any effect on linear and dichotomic outcome measures. These findings align with several recent works with different designs focused on p-tau181 and amyloid species [42–44], although the short follow-up and the exclusion of patients without dementia a priori definitively limit the number of patients at risk of conversion of this specific population.

These findings, in line with earlier reports, further highlighted the value of NfL as very sensitive yet unspecific marker of neuronal damage [45]. The clinical relevance of this marker alone—even when adjusting for clinical variables and adopting different models of progression—overshadowed all other more specific glial or AD-related markers in the cohort. These results are relevant for the research community, as they definitively questioned the added value of an extended panel of biomarkers for general stratification of PD instead of NfL alone [6, 33, 46–48]. Nevertheless, the study is exploratory in nature, as the population is lacking in ethnic and genetic diversity, as well as detailed kidney function, and further studies on different populations, comorbidity distributions, and longer follow-up should be conducted. Furthermore, studies with longitudinal assessment of plasma biomarkers changes are important to extend these findings. Another important limitation was the inclusion of consecutive PD patients who were already into the clinical phase of the disease, with a wide distribution of baseline disease duration and a relative stable disease, as highlighted by annual MDS-UPDRS changes lower compared with larger prospective studies [49]. To adjust for baseline severity, this variable was included in all multivariate models using clinically relevant outcome measures listed in PPMI and other prospective longitudinal PD studies [9]. Finally, further studies focused on drug-naïve PD or specific at-risk subpopulation (such as PD-MCI) are warranted to extend these findings and clarify the best combination and relevance of plasma biomarkers as proxies of disease progression in subjects suitable for interventions.

## Data Availability

The datasets used are available from the corresponding author on reasonable request.
